# An evidence-based knowledgebase of pulmonary arterial hypertension to identify genes and pathways relevant to pathogenesis†
†Electronic supplementary information (ESI) available. See DOI: 10.1039/c3mb70496c


**DOI:** 10.1039/c3mb70496c

**Published:** 2014-01-21

**Authors:** Min Zhao, Eric D. Austin, Anna R. Hemnes, James E. Loyd, Zhongming Zhao

**Affiliations:** a Department of Biomedical Informatics , Vanderbilt University School of Medicine , Nashville , TN , USA . Email: zhongming.zhao@vanderbilt.edu; b Department of Pediatrics , Vanderbilt University School of Medicine , Nashville , TN , USA; c Division of Allergy , Pulmonary and Critical Care Medicine , Vanderbilt University School of Medicine , Nashville , TN , USA; d Department of Medicine , Vanderbilt University Medical Center , Nashville , TN , USA; e Department of Cancer Biology , Vanderbilt University School of Medicine , Nashville , TN , USA; f Department of Psychiatry , Vanderbilt University School of Medicine , Nashville , TN , USA; g Center for Quantitative Sciences , Vanderbilt University Medical Center , Nashville , TN , USA

## Abstract

Pulmonary arterial hypertension (PAH) is a major progressive form of pulmonary hypertension (PH) with more than 4800 patients in the United States. In the last two decades, many studies have identified numerous genes associated with this disease. However, there is no comprehensive research resource for PAH or other PH types that integrates various genetic studies and their related biological information. Thus, the number of associated genes, and their strength of evidence, is unclear. In this study, we tested the hypothesis that a web-based knowledgebase could be used to develop a biological map of highly interrelated, functionally important genes in PAH. We developed the pulmonary arterial hypertension knowledgebase (PAHKB, http://bioinfo.mc.vanderbilt.edu/PAHKB/), a comprehensive database with a user-friendly web interface. PAHKB extracts genetic data from all available sources, including those from association studies, genetic mutation, gene expression, animal model, supporting literature, various genomic annotations, gene networks, cellular and regulatory pathways, as well as microRNAs. Moreover, PAHKB provides online tools for data browsing and searching, data integration, pathway graphical presentation, and gene ranking. In the current release, PAHKB contains 341 human PH-related genes (293 protein coding and 48 non-coding genes) curated from over 1000 PubMed abstracts. Based on the top 39 ranked PAH-related genes in PAHKB, we constructed a core biological map. This core map was enriched with the TGF-beta signaling pathway, focal adhesion, cytokine–cytokine receptor interaction, and MAPK signaling. In addition, the reconstructed map elucidates several novel cancer signaling pathways, which may provide clues to support the application of anti-cancer therapeutics to PAH. In summary, we have developed a system for the identification of core PH-related genes and identified critical signaling pathways that may be relevant to PAH pathogenesis. This system can be easily applied to other pulmonary diseases.

## Abstract 2

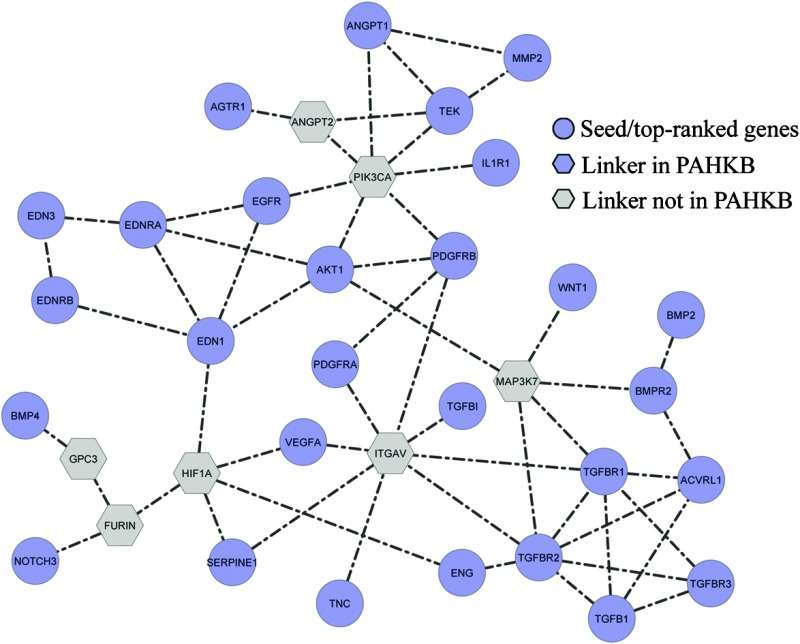
First literature-based, high-quality gene resource focused on pulmonary arterial hypertension (PAH) to identify genes and pathways relevant to PAH pathogenesis.

## Introduction

Pulmonary hypertension (PH) is the inappropriate elevation of pressure in the pulmonary vascular system.^1^ Pulmonary arterial hypertension (PAH) is a progressive form of PH characterized by pulmonary vascular remodeling of the distal pulmonary vasculature, ultimately leading to the destruction and loss of the smallest pulmonary arteries.^2^ The ensuing syndrome, PAH, is clinically characterized by reduced pulmonary arterial circulatory flow resulting in increased pulmonary vascular resistance, which ultimately results in the failure of the right heart and death.^3^

PAH has a high annual mortality rate despite recent progress and a surge of data generation with regard to the molecular understanding of this syndrome, such that a third of all patients still die within 3 years of diagnosis.^4,5^ As a result, improved understanding of the genetic and molecular risk factors in the pathogenesis of PAH represents a critical opportunity for the development of effective treatments. Because PAH represents one subtype of a larger syndrome of pulmonary vascular disease,^1^ and molecular advances in the field of PAH are often more widely applicable to other forms of pulmonary vascular disease, progress in the PAH research field often benefits a broader understanding of PH.

The pathology of PAH involves multiple processes/factors that influence vascular remodeling. In terms of the genetics of PAH, germline mutations in gene encoding bone morphogenetic protein receptor type 2 (*BMPR2*) are responsible for heritable PAH (HPAH) in 80–85% of families with PAH family history. Furthermore, 5–25% of patients diagnosed as having idiopathic PAH (IPAH) actually have a detectable germline mutation in *BMPR2* as well.^6–12^ Thus, *BMPR2* mutations constitute the largest known risk for developing PAH. However, recent studies have uncovered additional rare and common variants relevant to disease pathogenesis.^13–15^ With the rapid progress of high-throughput technologies, extensive basic and translational research has identified genes that may be associated with PAH development.^13,14,16,17^

A key challenge moving forward will be pinpointing how to integrate knowledge from different sources to prioritize key molecular pathways and generate testable hypotheses linked to personalized therapeutic interventions.^18–20^ In this study, we developed the first literature-based PAH data resource by comprehensively curating the literature data, importing high-throughput sequencing data, and gaining input from clinical experts. In the current release, the pulmonary arterial hypertension knowledgebase (PAHKB) contains 341 human PH-related genes (293 coding and 48 non-coding genes) curated from over one thousand PubMed abstracts. We demonstrated its application by constructing a core biological map of PAH. The online PAHKB interface, with browsing and searching functionalities, is available at ; http://bioinfo.mc.vanderbilt.edu/PAHKB/.

## Methods

### Extensive literature search for PAH-related genes

To provide a detailed and precise PAH-related gene resource supported by the literature, we first performed an extensive literature query comprising common components to study gene function: (“pulmonary arterial hypertension”[Title/Abstract] OR “IPAH”[Title/Abstract] OR “HPAH”[Title/Abstract] OR “pulmonary hypertension”[Title/Abstract]) AND ((“genome-wide association study” [Title/Abstract] OR “genome wide association study” [Title/Abstract]) OR (“gene”[Title/Abstract] AND (“association”[Title/Abstract] OR “microarray” [Title/Abstract] OR “expression” [Title/Abstract] OR “linkage” [Title/Abstract] OR “proteomics” [Title/Abstract] OR “genetic” [Title/Abstract] OR “metabolomics” [Title/Abstract] OR “copy number variation” [Title/Abstract] OR “idiopathic” [Title/Abstract] OR “hereditable” [Title/Abstract] OR “family” [Title/Abstract] OR “mouse model” [Title/Abstract] OR “animal model” [Title/Abstract] OR “microRNA” [Title/Abstract] OR “mutation” [Title/Abstract] OR “SNP” [Title/Abstract] OR “drug” [Title/Abstract] OR “transporter” [Title/Abstract]))). This complex query resulted in 911 PubMed abstracts on April 15th, 2013. Next, we extracted 516 sentences from 353 PubMed abstracts using “pulmonary” and “hypertension” as keywords from the Generif database^21^ on April 15th, 2013. Combining the two exhaustive searches, a total of 1161 PubMed abstracts were collected and downloaded in the Medline format for further curation.

### Data collection from literature

To collect a comprehensive gene list related to PAH, we manually curated PAH-related genes from literature sources using three major steps.^22,23^ We first grouped all 1161 PubMed abstracts by topic using the “Related Articles” function in the NCBI Entrez system. Next, we extracted PH-related descriptions from the grouped abstracts. Finally, we manually checked gene names and organism information extracted from the descriptions and mapped the gene names to NCBI Entrez human gene IDs.^24^ The primary aim of PAHKB is to collect and maintain a high quality PAH-related gene database, which serves as a comprehensive, fully classified, and accurately annotated PAH-related gene knowledgebase. In practical application, the genes related to other types of PH might increase our understanding of PAH. In addition, the gene content related to other PH types allowed us to assess if and how the collected PAH-related genes share molecular mechanisms with other types of PH and provided cross-checking between different gene sets. In total, we consolidated 341 human (293 coding and 48 non-coding genes, Table S1, ESI†) PH-related genes from 365 PubMed abstracts. According to literature evidence, we categorized these genes into three classes: 261 PAH-related genes, 29 genes with literature support to hypoxia pulmonary hypertension (HPH), and 121 genes related to other PH that were neither PAH nor HPH specific. As shown in Table S1 (ESI†), 10 genes were reported to be involved in all three PH subtypes: PAH, HPH, and other types of PH. These ten genes are: *BMP2*, *BMPR2*, *EDN1*, *HMOX1*, *NOS2*, *ROCK1*, *SMAD5*, *SLC6A4, TPH1*, and *TRPC6*.

Because of the importance of *BMPR2* in PAH, we collected *BMPR*-related mutations from a recently published comprehensive review,^25^ which can be found in our web browsing interface. To facilitate experimental access to existent animal models, we also collected all the mouse and rat knockout or abnormal expression models for PAH-related genes from literature. Finally, 53 animal models supported by the literature were included in our “Animal model” data set, which can be found at animal model page: ; http://bioinfo.mc.vanderbilt.edu/PAHKB/animalmodel.cgi.

### Annotation and database construction

To better understand the function of these collected PAH-related genes, we gathered extensive functional information from public data resources. The representative annotations in the PAHKB are summarized in Table 1. General gene information (such as gene symbol and synonyms) is integrated from the NCBI Entrez gene database.^24^ In addition, to provide the literature related to PAH for each gene, we also generated hyperlinks to the text mining server iHOP^26^ and the biomedical literature databases PubMed and GeneRIF.^21^ To help the user understand the biological pathways and involved diseases for each gene in PAHKB, we retrieved the pathway information from BioCyc,^27^ KEGG Pathway,^28^ PID Curated,^29^ PANTHER,^30^ and Reactome^31^ as well as possible disease associations with diseases from KEGG Disease,^28^ Fundo,^32,33^ GAD,^34^ NHGIR,^35^ and OMIM^24^ using the functional annotation server KOBAS.^36^ In addition, potential post-translational modifications, transcription factor regulation information, and genomic functional elements were collected from dbPTM,^37^ the TRANSFAC database,^38^ and the ENCODE RegulomeDB,^39^ respectively. Digital gene expressions for human PAH-related samples from GSE22356^40^ and lung development related samples from GSE14334^41^ were integrated from the Gene Expression Omnibus (GEO) database.^42^ Information about genomic variants, methylation sites, and protein–protein interactions were integrated from the COSMIC,^43^ DiseaseMeth,^44^ and Pathway Commons^45^ databases, respectively. All collected data are stored in a MySQL relational database.^22,46^

**Table 1:** Annotation entry statistics for 341 human pulmonary hypertension-related genes

Data category	Related entries	Annotated PH-related genes	Content/sources
General information			
Human PH-related genes	341	341	Gene symbol, synonym, genomics position, gene type from Entrez gene database
Literature	365	341	Curated literature evidence for PH-related genes

Function and regulation			
Pathway	3138	251	KEGG and HumanCyc database, *etc.*
Disease	5416	217	OMIM and GAD databases, *etc.*
Transcription factor regulation	5981	271	Regulatory reactions initiated by transcription factors from TRANSFAC
Post-translational modification	1451	199	Experimentally validated PTMs from dbPTM

Expression and methylation			
Gene expression	765	282	Expression in PAH-related samples from GSE22356 and lung development related samples from GSE14334
Methylation	1197	250	Methylation profiles in promoter regions from the DiseaseMeth database

Genomic variation			
Substitutions	8332	291	Single nucleotide mutations
Insertions/deletions	2151	36	Insertions and deletions
Other mutations	10 532	72	Others mutations

Functional interaction			
Physical interactions	22 764	254	Physical protein–protein interactions from high throughput data
Metabolic interactions	446	72	Connected metabolic reactions
Signaling interactions	7349	150	Consecutive signaling transduction

### Biological functional analysis and network-based analysis

To evaluate the functional significance of the interesting genes, we performed functional enrichment analyses on the KEGG canonical pathways and Gene Ontology (GO) terms using WebGestalt (WEB-based GEne SeT AnaLysis Toolkit).^47^ To assess the protein domain of the interesting gene sets, we conducted hypergeometric enrichment tests using the online tool DAVID (Database for Annotation, Visualization and Integrated Discovery).^48^ For all these functional analyses, we chose those significant terms with an adjusted *p*-value of less than 0.05 as calculated by the hypergeometric test followed by the Benjamini–Hochberg method for multiple testing correction,^49^ which were steps implemented in the WebGestalt and DAVID tools.

To form a connected pathway for the 261 PAH-related genes, we first constructed a non-redundant human interactome based on the consecutive metabolic reactions and signaling transduction data from HumanCyc, NCI signaling pathway database, Reactome, and Cell-map pathway databases. It is noteworthy that the collected gene–gene interaction relationship is different from high-throughput protein–protein interactions, which are physical interactions without biological significance. The final interactome contains 3629 nodes and 36 034 pathway-based gene–gene interaction links. To extract a sub-network related to the 39 PAH-related genes of interest, we used the Steiner minimal tree algorithm implemented in GenRev toolkits.^50^ In this algorithm, all inputted genes were mapped to the pathway-based interactome. Finally, a minimum sub-network with inputted genes connected by shortest path was produced. The final network visualization was performed using Cytoscape.^51^

## Results

### Typical gene entry in PAHKB

**Fig. 1:**
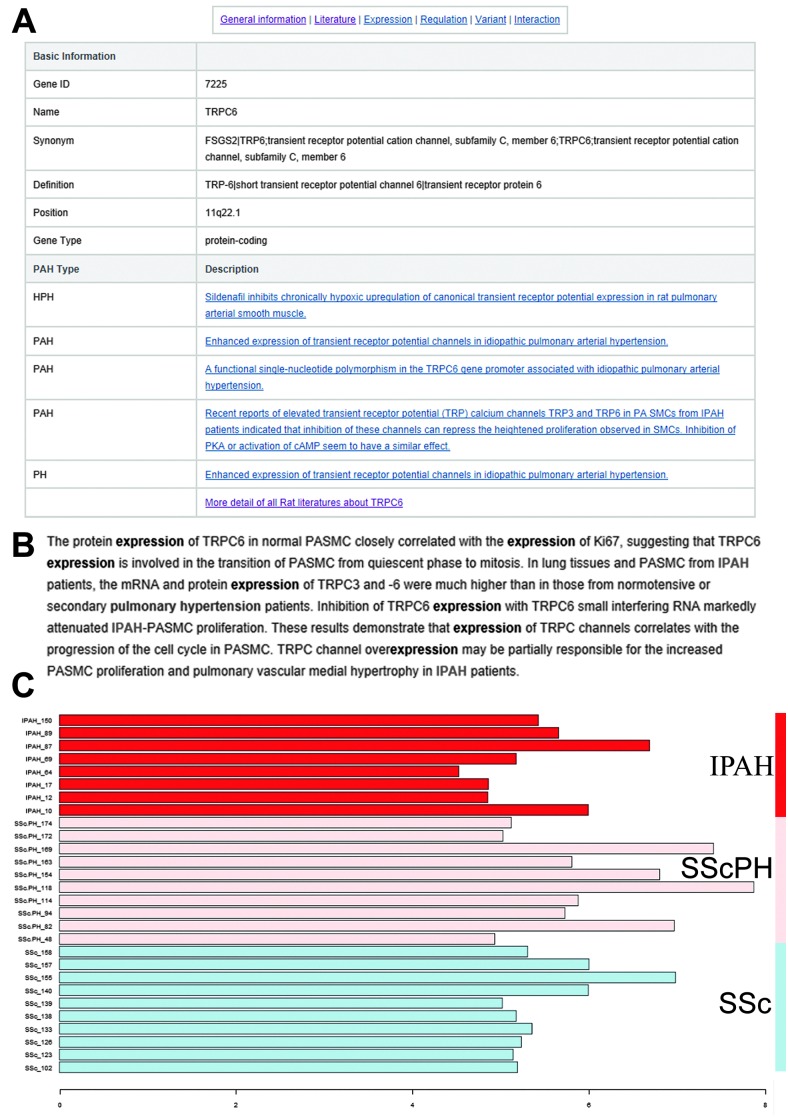
Gene information in the PAHKB database. (A) Basic gene information in the PAHKB database. (B) A typical highlighted literature with supporting keywords. (C) Gene expression profile. IPAH: idiopathic pulmonary arterial hypertension, SScPH: scleroderma-related pulmonary hypertension, SSc: scleroderma sample.

**Fig. 2:**
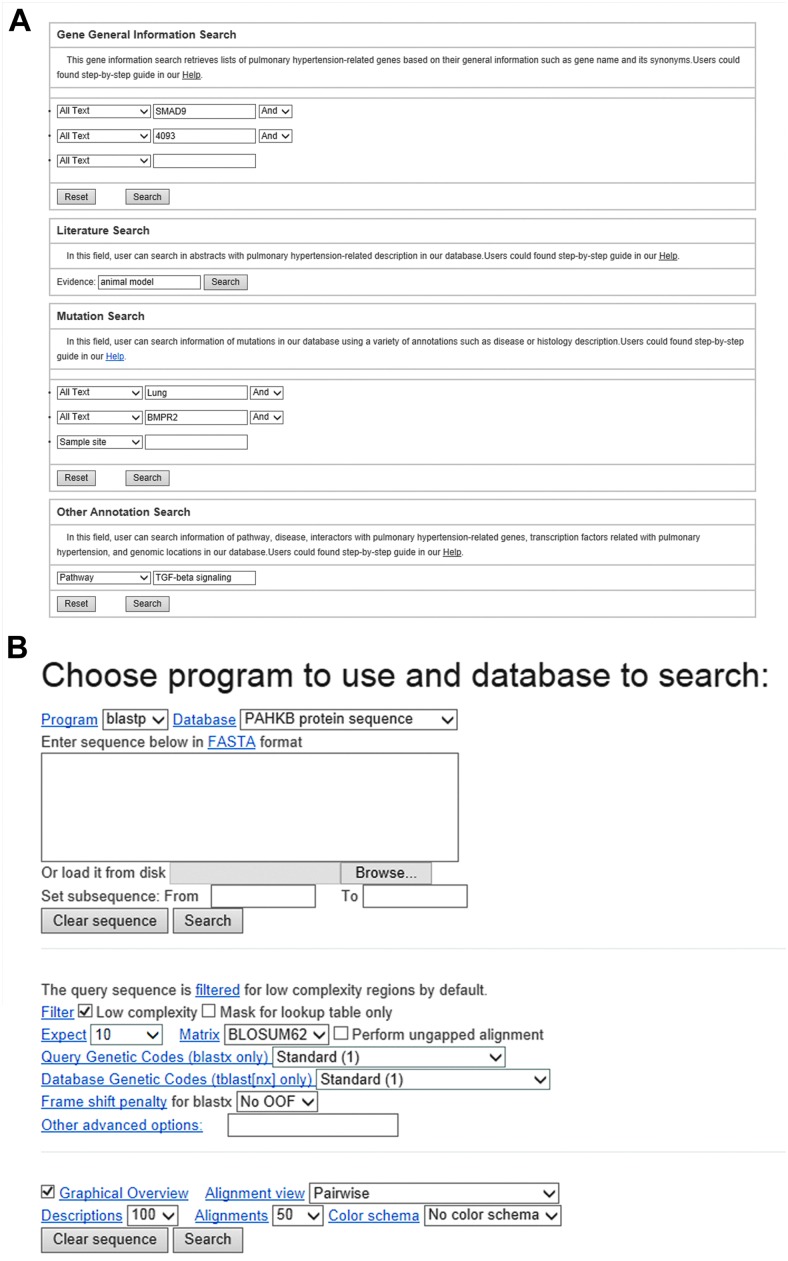
An interface for searching data from the PAHKB database. (A) Keyword-based query interface. (B) Sequence search *via* the BLAST interface.

**Fig. 3:**
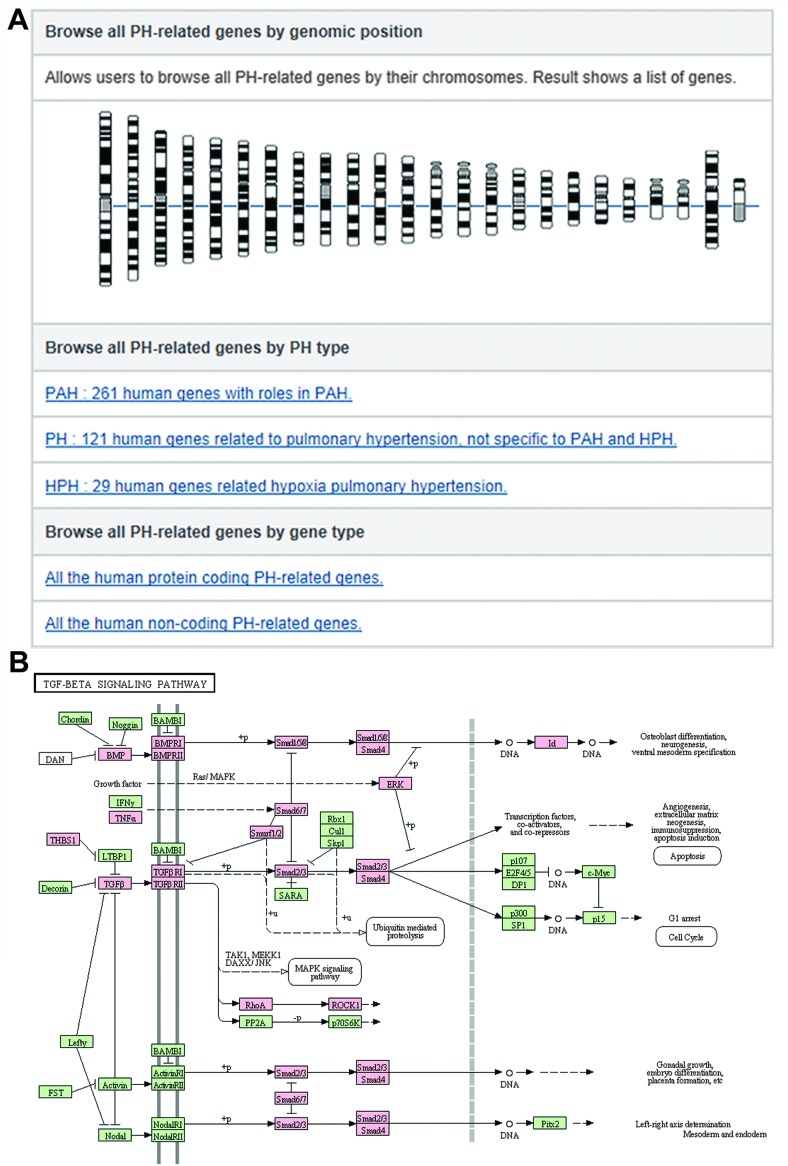
An interface for browsing data from the PAHKB database. (A) Browsing PH-related genes by chromosome location, disease type, and genic region (protein-coding or non-coding region). (B) An example of browsing the data by pathway: KEGG TGF-beta signaling pathway mapped with PAH-related genes (color-marked) in the PAHKB database.

As shown in Fig. 1–3, the annotations of a typical PAHKB gene entry can be categorized into six types: “General information,” “Literature,” “Expression,” “Regulation,” “Mutation,” and “Interaction.” By clicking on “General information” in each gene page, the user can access the gene name, involved pathways and diseases, nucleotide sequence, and protein sequence in a tabular view (Fig. 1A). Highlighted summaries from the curated literature are provided in “Literature” (Fig. 1B). In “Expression,” gene expressions from PAH-related samples and lung development-related samples are provided as a bar graph with accompanying sample names and normalized expression scores listed (Fig. 1C). This bar graph is useful to acquire an overview of the expression specificity of each PAH-related gene among different disease types and lung developmental stages.

### Web interface of PAHKB to search, browse, and download data

Both text query and sequence search capabilities are provided to access the PAHKB. On the top right of each web page, users can perform a quick text search using either the Entrez gene symbol or gene ID. A more complex text query interface is also available in order to search based on the gene symbol, Entrez gene ID, genomic location, disease, and pathway. Furthermore, we also provide a query interface to access all the curated literatures in PAHKB, which allows users to find more comprehensive PAH-related gene descriptions from original literature sources (Fig. 2A). Moreover, the user can utilize an online BLAST interface to search against all PAH-related genes through their nucleotide or protein sequences by inputting an interesting sequence in a FASTA format (Fig. 2B). We also provide bulk downloads of data for advanced bioinformatics users to further systematically analyze.

The PAHKB also provides browsing functions for different data sources, disease subtypes, graphically represented pathways, protein-coding and non-coding genes, and genomic locations (Fig. 3). In the disease type browser page, users can click on the hyperlinks for specific PH subtypes to view all the reported PAH-related genes, HPH-related genes, and other PH-related genes with literature evidence connected to each subtype (Fig. 3A). Through the chromosome browser, users can obtain PH-related gene lists that include a summary of the genes as well as hyperlinks to detailed evidence and annotation pages (Fig. 3A). In addition, users can explore 58 human KEGG pathways with any human PH-related genes in striking color (Fig. 3B). Clicking on the highlighted PH-related genes in the KEGG pathway map allows users to access corresponding entries in our database.

### Enriched biological pathways for 261 PAH-related genes

To better understand the function of these PAH-related genes in our database, we performed pathway enrichment and disease association analyses on the 261 PAH human genes to obtain general insights into their biological features using the WebGestalt server. Over-represented pathways and significantly associated diseases were determined by an adjusted *p*-value of less than 0.05 calculated using the hypergeometric test followed by the Benjamini–Hochberg multiple testing correction.^36^ As shown in Table 2, the enriched KEGG pathways include signaling pathways (“TGF-beta signaling pathway,” “Wnt signaling pathway,” “MAPK signaling pathway,” *etc.*), extracellular interaction pathways (“Cytokine–cytokine receptor interaction,” “Focal adhesion,” *etc.*) and cancer signaling pathways (“Pathways in cancer,” “Pancreatic cancer,” *etc.*). To further assess the functional distribution of GO terms and protein domains, we conducted enrichment tests on the 261 human PAH-related genes. We selected those GO terms or protein domains with an adjusted *p*-value of less than 0.05 as calculated using the hypergeometric test followed by the Benjamini–Hochberg correction.^49^ Using the complete human gene list as the background, according to the GO database, the 261 protein-coding PAH human genes were over-represented in processes that included cell proliferation, locomotion, regulation of biological quality, and developmental process (Fig. S1, ESI†). In addition, the most frequently represented InterPro domains were “Protein kinase, core,” “TGF-beta receptor/activin receptor, type I/II,” “Short-chain dehydrogenase/reductase SDR,” “MAD homology, MH1,” “SMAD domain-like,” and “SMAD domain, Dwarfin-type.” These results highlight the fundamental roles that signaling transduction of PAH-related genes play in controlling cell proliferation (Table S3, ESI†).

**Table 2:** Top 20 enriched KEGG pathways with the 261 PAH-related genes

KEGG pathway	*p*-Value	Benjamini–Hochberg corrected *p*-value
Pathways in cancer	4.61 × 10^–37^	5.30 × 10^–35^
Cytokine–cytokine receptor interaction	3.13 × 10^–33^	1.80 × 10^–31^
TGF-beta signaling pathway	6.52 × 10^–32^	2.50 × 10^–30^
Rheumatoid arthritis	8.92 × 10^–21^	2.56 × 10^–19^
Focal adhesion	6.15 × 10^–20^	1.41 × 10^–18^
Pancreatic cancer	1.91 × 10^–19^	3.66 × 10^–18^
Toxoplasmosis	6.70 × 10^–18^	1.10 × 10^–16^
Colorectal cancer	6.90 × 10^–17^	9.92 × 10^–16^
Osteoclast differentiation	2.57 × 10^–15^	3.28 × 10^–14^
Chagas disease (American trypanosomiasis)	3.05 × 10^–15^	3.51 × 10^–14^
MAPK signaling pathway	5.66 × 10^–15^	5.92 × 10^–14^
Leishmaniasis	2.09 × 10^–14^	2.00 × 10^–13^
Prostate cancer	2.94 × 10^–13^	2.60 × 10^–12^
Gap junction	3.38 × 10^–13^	2.78 × 10^–12^
Viral myocarditis	5.10 × 10^–13^	3.86 × 10^–12^
Wnt signaling pathway	5.37 × 10^–13^	3.86 × 10^–12^
Steroid hormone biosynthesis	1.55 × 10^–12^	1.05 × 10^–11^
Calcium signaling pathway	5.20 × 10^–12^	3.32 × 10^–11^
Chemokine signaling pathway	1.26 × 10^–11^	7.63 × 10^–11^
Adherens junction	2.46 × 10^–11^	1.35 × 10^–10^

### Gene prioritization for PAH-related genes

To help the user evaluate the importance of each gene in PAHKB, we performed gene prioritization using the Endeavour web server.^52^ Endeavour integrates multiple genomic data sources to rank the candidate genes, including functional annotations, protein–protein interactions, regulatory information, expression data, sequence based data, and literature mining data. Endeavour requires two inputs: training genes and candidate genes. The training dataset contains genes already known to play an important role in PAH. Starting with the training genes, Endeavour first builds relevant importance from each genomic data source. Then, it utilizes the resulting relative importance from all data sources for gene prioritization. In the present study, we compiled a core gene list that included 9 genes (*BMPR2*, *SLC6A4*, *EDN1*, *ACVRL1*, *NPPB*, *ENG*, *TEK*, *KCNA5*, and *ACE*) with robust literature evidence in PAH to build a scoring model. In the second stage, the scoring model was used to rank the candidate genes for each genomic data source. Finally, Endeavour combined all the ranking scores, creating a global ranking for all the input PH-related genes using order statistics. In total, 209 valid human genes were ranked (Table S2, ESI†). The top ten ranked genes were *BMPR1A*, *TGFBR1*, *TGFBR2*, *ANGPT1*, *PDGFRA*, *PDGFRB*, *TGFBR3*, *SCN5A*, *SERPINE1*, and *TGFB1*. Not surprisingly, the majority of these top ranked genes are involved in key pathways of PAH, such as the “TGF-beta signaling pathway.” Although these candidate genes have been demonstrated to have abnormal gene expression or other functional relevance to PAH, most of them have not been reported as having detectable genetic variants in PAH patients.

### Constructing a core biological pathway based on the highly ranked PAH-related genes

**Fig. 4:**
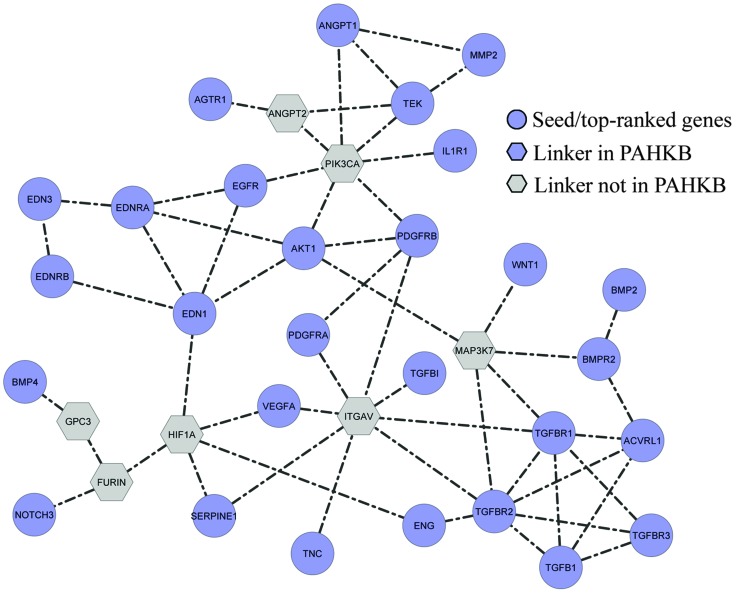
Constructed biological map for PAH-related genes from pathway-based interaction data. The blue circles (28 genes) are those from the input 39 top-ranked PAH-related genes. The grey hexagons (6) are the linker genes not in PAHKB. The gene HIF1A (blue hexagon) is a linker gene in our PAHKB but not in the input 39 top-ranked PAH-related genes.

To further explore the biological meaning of the prioritized PAH-related genes, we mapped the top 30 ranked genes and the 9 genes in the training dataset to the pathway-based protein interaction network. Next, we adopted a systems biology approach to reconstruct potential biological processes based on existing pathway databases using the 39 most important PAH-related genes as seeds. As a result, a sub-network consisting of 35 nodes and 55 edges was extracted. Among the 35 nodes, 28 were from the input 39 top-ranked PAH-related genes. The remaining 7 genes were used as links to help the 28 PAH-related genes form a well-connected network; these genes are referred to as “linker genes.” Among the 7 linker genes, *HIF1A* is related to PAH and was included in our PAHKB. This sub-network represents the predicted biological pathway centered by 39 PAH-related genes (Fig. 4). The follow-up KEGG pathway enrichment analysis on the 35 genes (Table 3) displayed a similar functional distribution to that of all the 261 PAH-related genes (Table 2). Most of the pathways are related to known pathways such as “TGF-beta signaling pathway,” “Focal adhesion,” “Cytokine–cytokine receptor interaction,” and “MAPK signaling pathway.” However, there are a few cancer signaling pathways that are enriched in both the 261 PAH-related genes and our constructed core biological pathway for PAH.

**Table 3:** Top 10 KEGG pathways enriched with the genes in the biological map constructed by top-ranked PAH-related genes

KEGG pathway	*p*-Value	Benjamini–Hochberg corrected *p*-value
Pathways in cancer	3.08 × 10^–23^	1.72 × 10^–21^
Cytokine–cytokine receptor interaction	1.04 × 10^–14^	2.91 × 10^–13^
Pancreatic cancer	1.41 × 10^–13^	2.63 × 10^–12^
MAPK signaling pathway	7.41 × 10^–13^	1.04 × 10^–11^
Focal adhesion	3.93 × 10^–12^	4.40 × 10^–11^
Osteoclast differentiation	1.08 × 10^–11^	1.01 × 10^–10^
TGF-beta signaling pathway	7.06 × 10^–11^	5.65 × 10^–10^
Chagas disease (American trypanosomiasis)	2.61 × 10^–10^	1.83 × 10^–09^
Colorectal cancer	1.64 × 10^–09^	1.02 × 10^–08^
Glioma	2.08 × 10^–09^	1.16 × 10^–08^

## Discussion

In this study, we developed the first literature-based PAH genetic resource, which currently contains 341 human genes extracted by comprehensively curating the literature data, importing high-throughput sequencing data, and gaining input from clinical experts. PAHKB is the first attempt to establish a literature-based knowledgebase of PAH with a user-friendly web interface, which provides users with a sophisticated text query, sequence search, gene ranking, browsing using functional analysis results, a highlighted pathway map, and curated mouse model. The typical queries include gene information, literature evidence, known mutation, and functional annotation.

To test the PAHKB, we applied an integrative systems-based approach to rank PAH-related genes and form network-based functional analyses.^53,54^ The results support both previously known and novel gene networks related to PAH. For example, we uncovered pathways relevant to PAH that are highly relevant to cancer pathogenesis as well. This is not surprising, as the cancer paradigm of PAH has recently been an area of intense interest in the field.^55^ It is now believed that a hallmark of the vascular obliteration in PAH includes endothelial cell proliferation that is not balanced by adequate apoptosis. This abnormal cell proliferation results in progressive pulmonary vascular disease that is not targeted by current therapies. Our integrative analysis may advance the understanding the cellular factors that promote proliferative PAH, which may elucidate novel pathways for future drug development.

PAHKB can be used for multiple purposes, including: (i) obtaining literature-based and importance ranked gene lists for PAH and other types of PH; (ii) reviewing comprehensive annotations, including regulatory features from ENCODE data, involved biological pathways, protein–protein interactions, methylation sites, transcription factor neduated regulation, and post-translational modification; and, (iii) a resource for high-throughput genetic and clinical tests to find PAH-related genetic variants. Overall, our curated PAH-related gene list maps the genomic and cellular landscape for PAH-related genes, providing a valuable resource for the PH research community.

With the rapid increase in advanced gene and expression assays at high-throughput levels, the volume of data published related to PH and PAH continues to expand. While the future of personalized medicine in pulmonology and cardiology will include a systems biology approach, there is great opportunity at the population level as well. Complex genetic and genomic alterations may occur due to a wide variety of variants, including common variants, rare variants (mutations), and epigenetic phenomena. A systems biology approach will be necessary to integrate large volumes of data and determine the network of interactions, at the cellular level, that regulate activity as well as ultimately associate with disease phenotypes. At first glance, one might conclude that our initial test of the PAHKB simply identified the pre-existing known pathways in PAH; however, deeper analysis showed substantially more information. The analytic approach made possible by the PAHKB allowed us to quickly identify the gaps between known genes and pathways, which will provide novel targets for future study. For example, the ‘Linker’ nodes in Fig. 4 represent genes and proteins that are vital to the core biologic pathway of PAH but have yet to be described in the literature. Thus, a free and open multimodal system that integrates DNA, RNA, microRNA, methylation, proteomics, metabolomics, and other resources with the ability for continual updates should provide a significant resource to the PAH researcher community. Interpretation of our study relies on the reliable candidate gene list for PAH from the literatures. With more large-scale gnomic data, the integrative based approach will play more important roles to discover novel pathogenetic mechanisms. We will update the PAHKB database periodically through regular literature search, expert review, extraction of data from large-scale datasets (*e.g.* genomic data), among others. The update information will be provided on PAHKB website.

## Conclusions

We have developed an online genetic resource to record core PH-related genes and identify critical signaling pathways that may be relevant to PAH pathogenesis. This computational system can be easily applied to other pulmonary diseases and is useful resource to pulmonary research community.
