# Supplementary material for: An evidence-based knowledgebase of pulmonary arterial hypertension to identify genes and pathways relevant to pathogenesis
Source: Mol Biosyst. 2014 Jan 21;10(4):732–40. doi: 10.1039/c3mb70496c (PMC3950334; doi:10.1039/c3mb70496c)

Electronic Supplementary Material (ESI) for Molecular BioSystems
This journal is © The Royal Society of Chemistry 2014

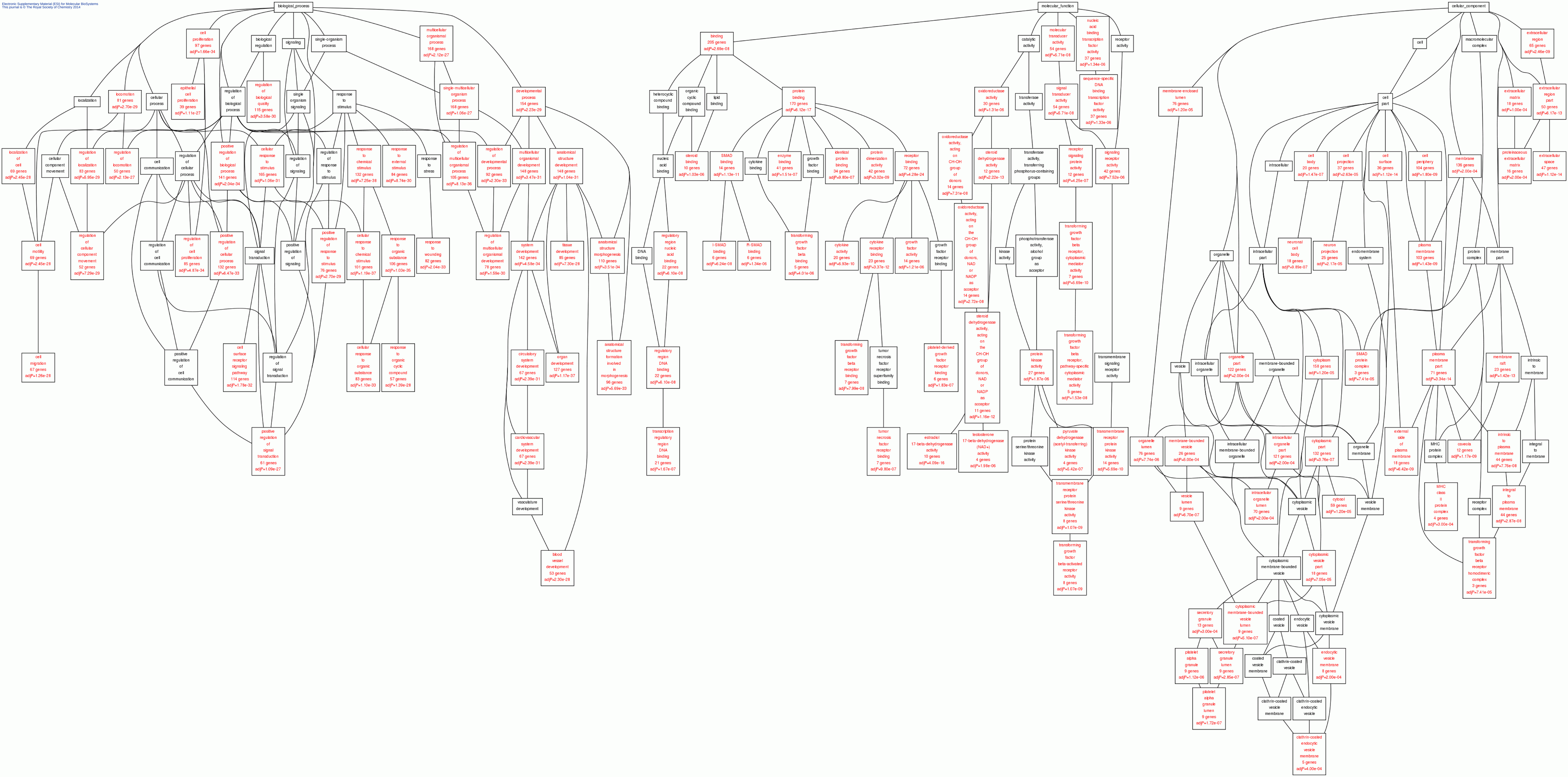

Supplement: Supplementary file 1 [file MB-010-C3MB70496C-s001.pdf]
